# Quality by Design (QbD)-Based Development of a Self-Nanoemulsifying Drug Delivery System for the Ocular Delivery of Flurbiprofen

**DOI:** 10.3390/pharmaceutics17050629

**Published:** 2025-05-09

**Authors:** Ju-Hwan Jeong, Tae-Han Yoon, Si-Won Ryu, Min-Gyeong Kim, Gu-Hae Kim, Ye-Jin Oh, Su-Jeong Lee, Na-Woon Kwak, Kyu-Ho Bang, Kyeong-Soo Kim

**Affiliations:** Department of Pharmaceutical Engineering, Gyeongsang National University, 33 Dongjin-ro, Jinju 52725, Republic of Korea; wnghks0810@naver.com (J.-H.J.); xogks7702@naver.com (T.-H.Y.); rsiwon0316@gnu.ac.kr (S.-W.R.); als7573140@naver.com (M.-G.K.); rlarngo1052@gmail.com (G.-H.K.); zioune05@gmail.com (Y.-J.O.); dltnwjd9148@naver.com (S.-J.L.); skdns9945@gmail.com (N.-W.K.); khbang0095@gnu.ac.kr (K.-H.B.)

**Keywords:** self-nanoemulsifying drug delivery system, flurbiprofen, Box–Behnken design, ocular delivery, quality by design, nanoemulsion

## Abstract

**Objectives**: In this study, Quality by Design (QbD) was used to develop an optimized self-nanoemulsifying drug delivery system (SNEDDS) as an ophthalmic formulation of flurbiprofen (FLU). Using a Box–Behnken design (BBD), an optimal SNEDDS composition was crafted, targeting enhanced corneal permeability and increased bioavailability of the drug. **Methods**: The levels of each factor(X) were established using a pseudo-ternary diagram, and the Box-Behnken design (BBD) was used to evaluate the components of oil (18.9 mg), surfactant (70.7 mg), and co-surfactant (10.0 mg) to optimize the SNEDDS formulation. The response(Y) considered were particle size, polydispersity index (PDI), transmittance, and stability. Transmission electron microscopy (TEM) and dynamic light scattering (DLS) were used to analyze the particle size and morphology. In vitro and ex vivo diffusion tests were conducted to assess drug flux and permeability. **Result**: Using a response optimization tool, the values of each X factor were optimized to achieve a small particle size (nm), a low polydispersity index (PDI), and high transmittance (%), resulting in a formulation prepared with 18.9 mg of oil, 70.7 mg of surfactant, and 10.0 mg of co-surfactant. The optimized SNEDDS exhibited a small particle size of 24.89 nm, a minimal PDI of 0.068, and a high transmittance of 74.85%. A transmission electron microscopy (TEM) analysis confirmed the presence of uniform spherical nanoemulsion droplets with an observed mean diameter of less than 25 nm, corroborating the dynamic light scattering (DLS) measurements. Furthermore, the SNEDDS demonstrated improved stability under the stress conditions of heating–cooling cycles, with no phase separation, creaming, or caking observed and no differences in its particle size, PDI, or transmittance. In vitro and ex vivo diffusion tests demonstrated that the flux of the optimized SNEDDS (2.723 ± 0.133 mg/cm^2^, 5.446 ± 0.390 μg/cm^2^) was about 2.5 and 4 times higher than that of the drug dispersion, and the initial diffusion was faster, which is suitable for the characteristics of eye drops. **Conclusions**: Therefore, the formulation of a flurbiprofen-loaded SNEDDS (FLU-SNE) was successfully optimized using the QbD approach. The optimized FLU-SNE exhibited excellent stability and enhanced permeability, suggesting its potential effectiveness in treating various ocular inflammations, including uveitis and cystoid macular edema.

## 1. Introduction

The role of the uvea in human vision is essential because it provides numerous blood vessels that nourish various parts of the eye. Postoperative inflammation is usually characterized by severe inflammation affecting the uvea, and any inflammatory disease that damages the uvea can lead to visual impairments [1]. The topically applied nonsteroidal anti-inflammatory drug (NSAID) flurbiprofen is used as an ocular therapeutic agent for conditions such as acute cystoid macular edema (CME) or uveitis following cataract surgery [2,3,4]. It can also help reduce ocular surgery times by preventing the release of inflammatory mediators in the anterior segment [5]. A significant challenge in delivering therapeutics for ocular diseases is the limited topical absorption of the applied drug, which is primarily because of the impermeable cornea [6,7]. Although poorly water-soluble drugs formulated as eye drops have the advantage of being able to cross the lipophilic cornea, their limited aqueous solubility is a major factor limiting their therapeutic potential because of their low ocular bioavailability [8]. Self-nanoemulsifying drug delivery systems (SNEDDSs) with uniform preconcentrations of oil, surfactants, and co-surfactants have been employed to enhance the solubility of poorly water-soluble drugs by forming o/w nanoemulsions upon dilution [9,10,11,12]. SNEDDSs represent a novel approach that warrants more attention in the ocular field as an advanced in situ generator of a nanoemulsion [13]. An SNEDDS can spontaneously undergo a fine emulsification process in aqueous environments, such as the gastrointestinal tract, even with mild agitation, forming a nano-sized emulsion with a dispersed particle size generally remaining below 200 nm [14]. This small particle size increases the interfacial surface area for drug absorption, which enhances drug permeability [12,15,16]. FLU, a poorly water-soluble anti-inflammatory drug with a carboxyl functional group and a pKa of 4.23, belongs to Class II of the Biopharmaceutics Classification System (BCS) and is characterized by poor water solubility [17]. The objective of this study was to develop an optimal ophthalmic SNEDDS formulation containing FLU with a small particle size, a low PDI, and high transmittance using a Quality by Design (QbD) approach. The levels of oil, surfactant, and co-surfactant were established using a pseudo-ternary phase diagram, and the final formulation was optimized using a Box–Behnken response surface design [9]. SNEDDSs are non-irritating to the eyes, and FLU, Kollisolv MCT 70, Solutol HS 15, and Labrafil M1944 CS, used in FLU-SNE, are safe for the body, are non-irritating, and exhibit low toxicity [18,19,20,21,22,23]. The optimized formulation was subjected to characterization, including particle size, PDI, zeta potential, transmittance, pH, heating–cooling cycle, transmission electron microscopy (TEM), and Franz diffusion cell analyses. The membrane permeabilities of FLU-SNE and a drug dispersion were confirmed through in vitro membrane permeability experiments using cellulose membranes and ex vivo membrane permeability experiments using bovine corneas. To the best of our knowledge, there have been only a few studies on FLU-loaded SNEDDSs, and none of them reported an optimized formulation using QbD. Therefore, this work can be considered a novel contribution to the field of nanotechnological applications. We effectively optimized ophthalmic FLU-SNE by applying a BBD to develop a formulation with enhanced solubility, stability, and bioavailability, demonstrating the reproducibility, stability, and high permeability of the optimal formulation [12,16,24].

## 2. Materials and Methods

### 2.1. Materials

FLU was provided by Hanmi Pharmaceutical (Seoul, Republic of Korea). A phosphate-buffered saline (PBS) solution (pH 7.4) (WELGENE; Precision Solution, Gyeongsan, Republic of Korea), MCT oil, linseed oil, olive oil, castor oil, cotton seed oil, glycerol, mineral oil, and polysorbate 80 (Tween^®^ 80) were obtained from SAMCHUN GROUP (Daegu, Republic of Korea). Propylene glycol dicaprylate/dicaprate (Labrafac PG), oleoyl macrogolglycerides (Labrafil M2125CS), oleoyl polyoxyl-6 glycerides (Labrafil M1944CS), and propylene glycol monocaprylate (Capryol^®^ 90) were acquired from Gattefosse (St. Priest, France). Sunflower seed oil, polysorbate 20 (Tween^®^ 20), potassium phosphate monobasic, phosphoric acid, and sodium lauryl sulfate (SLS) were acquired from Daejung Chemicals (Siheung, Republic of Korea). Polyoxyl 15 hydroxystearate (Solutol HS 15), polyethoxylated castor oil (Cremophor EL), D-α-tocopheryl polyethylene glycol 1000 succinate (TPGS), polyoxyl 40 hydrogenated castor oil (Cremophor RH 40), Poloxamer 124 (Kollisolv P124), Poloxamer 407 (Kolliphor P407), and Poloxamer 188 (Kolliphor P188) were acquired from BASF (Ludwigshafen, Germany). Acetonitrile was purchased from Thermo Fisher Scientific (Waltham, MA, USA). A 12,400 Da cellulose membrane for a Franz diffusion cell (DHC-6TD, LOGAN Inc., Huntington, WV, USA) dialysis tube was purchased from Spectrum Life Sciences, LLC (SL Science, Hanam, Republic of Korea). The other chemicals used were of the appropriate class for the analysis.

### 2.2. HPLC Conditions

The HPLC analysis of the FLU samples was conducted using an Agilent 1260 Infinity HPLC system (Agilent Technologies, Santa Clara, CA, USA) equipped with a UV–Vis detector (Aglient G1314 1260, Agilent Technologies, Santa Clara, CA, USA). FLU was separated using a reversed-phase column (VDSpher PUR 100 C18 M-E; 5 μm; 4.6 × 150 mm; VDS optilab, Berlin, Germany). The mobile phase was composed of a solution containing mobile phase A (0.05 M potassium phosphate monobasic solution) and mobile phase B (acetonitrile) in a ratio of 60:40 (*v*/*v*). The HPLC analysis was performed at a flow rate of 1.0 mL/min. The injection volume was 10 μL, and UV detection was monitored at 222 nm [15]. Data acquisition and processing were performed using Agilent OpenLAB CDS Chemstation software LC software (Product Version: 2.18.18).

### 2.3. Solubility Test

We measured the solubility of FLU in various excipients such as oils, 10% (*w*/*v*) surfactants, and 10% co-surfactants. We added 10 mg of the drug to 1 mL of each surfactant, co-surfactant, and oil [25,26]. The solution containing the drug was mixed using a vortex mixer (VORTEX-2 GENIE, Scientific industries, New York, NY, USA) and stored in a shaking water bath (LSB-045S, Daehan Labtech, Namyangju, Republic of Korea) for 5 days at 37 °C and 50 rpm. The sample was centrifuged at 13,500 rpm for 15 min using a centrifuge (1730R, Gyrozen, Gimpo, Republic of Korea). The supernatant solution was obtained from the mixture, passed through a 0.45 μm syringe filter, and diluted with a mixture of mobile phases A and B. The amount of FLU was analyzed using the HPLC system conditions as described in Section 2.2. The experiments were repeated three times.

### 2.4. Pseudo-Ternary Phase Diagrams

Solubility test results were obtained to identify self-emulsifying areas under stirring conditions using a pseudo-ternary phase diagram. Based on the solubility test results for FLU, Kollisolv MCT 70, Solutol HS 15, and Labrafil M1944CS were selected as the oil, surfactant, and co-surfactant, respectively. A solution containing 0.2 mL of each composition was added to a beaker containing 200 mL D.W. at a temperature of 37 °C and magnetically stirred [27]. When these mixtures were visually observed, the region forming a nearly transparent and homogeneous emulsion without phase separation was classified as the ‘good’ region, whereas the region forming a turbid emulsion was classified as the ‘bad’ region [28,29,30]. We added 24.2 mg of FLU, equivalent to the commercially available B&L Flurbiprofen Sodium Ophthalmic solution 0.03%^®^, to the SNEDDS formulation corresponding to the ‘good’ region. The mixture was then stirred to verify the dissolution. We added 0.2 mL of the drug-dissolved SNEDDS to 200 mL of distilled water at a temperature of 37 °C. The nanoemulsion was judged to be a failure if we observed a turbid mixture or no emulsification progress. A pseudo-ternary phase diagram was generated using SigmaPlot v10 (Systat Software Inc., San Jose, CA, USA) and constructed based on the obtained self-emulsification observations.

### 2.5. Optimization of the FLU-SNE Formulation Using a Box–Behnken Design (BBD) Approach

Based on the ‘good’ area identified in the pseudo-ternary phase diagram described in Section 2.4, the FLU-SNE formulation was optimized using a Box–Behnken design and Minitab19^®^ (Minitab Inc., State College, PA, USA). The X factors were defined as X_1_ for oil, X_2_ for surfactant, and X_3_ for co-surfactant, with each concentration set at three levels: low (−1), medium (0), and high (+1) (Table 1). In total, 15 runs were performed by the software to estimate the error values, including three center points. The amount of FLU was constantly maintained at 2.4 mg for all 15 runs. The types of oil (Kollisolv MCT 70), surfactant (Solutol HS 15), and co-surfactant (Labrafil M1944CS) were consistent for all experiments. Based on the values selected as the good region in the pseudo-ternary phase diagram, the concentration of Kollisolv MCT 70 was set to 10–20 mg, that of Solutol HS 15 to 40–80 mg, and that of Labrafil M1944CS to 10–50 mg. The main effects and interaction effects were checked to confirm the particle size (nm) (Y_1_), PDI (Y_2_), zeta potential (mV) (Y_3_), and transmittance (%) (Y_4_). An ANOVA was used to assess the significance of each factor and determine the *p*-values for all responses. The most appropriate models were then selected based on the significant *p*-values obtained from the R^2^ analysis for each response variable.

### 2.6. Response Optimization and Design Space Development

Using the Box–Behnken Design (BBD) of the Response Surface Methodology (RSM), the relationships between the response and multiple X factors were identified. To determine the optimal conditions for the input variables, all responses must be considered simultaneously, which is referred to as a multiresponse problem. To develop the FLU-SNE formulation with the smallest particle size, the lowest PDI, and the highest transmittance, the multiresponse optimization tool in Minitab was used to determine the appropriate values of the X factors. Subsequently, the design space for optimizing the formulation variables was established, defining the range that provides the optimal product characteristics.

### 2.7. Particle Size and Zeta Potential Analyses

A sample of the optimized FLU-SNE formulation was diluted 10-fold in PBS. The particle size and PDI were determined using a Zetasizer Nano ZS (Malvern Inc., Malvern, UK) at a wavelength of 633 nm and a scattering angle of 173° [10]. The temperature was set to 37 °C. The hydrodynamic diameter was measured in triplicate. Zeta potential measurements were performed using a capillary cell (DTS 1070) and phase analysis light scattering (PALS), and the zeta potential was calculated using Smoluchowski’s equation. Measurements were conducted in triplicate.

### 2.8. pH Test

A sample of the optimized FLU-SNE formulation was diluted 10-fold in PBS. The pH of the sample was measured at 25 ± 0.5 °C using a pH meter (pH meter S210, Mettler-Toledo, Columbus, OH, USA) with a glass electrode.

### 2.9. Transmission Electron Microscopy

The morphology and particle size of the dispersed particles of FLU-SNE in a pH 6.8 buffer were analyzed using a transmission electron microscope (Talos L120C, Thermo Fisher Scientific Inc., Waltham, MA, USA). FLU-SNE was diluted 10-fold in PBS, dispensed dropwise onto a carbon-coated TEM grid (FCF200-CU-50, Electron Microscopy Sciences, Inc., Hatfield, PA, USA), and dried for 30 min in a desiccator. The prepared sample was then scanned at an accelerating voltage of 300 kV [26].

### 2.10. Heating and Cooling Cycles

FLU-SNE was subjected to six cycles of storage at 4 °C (refrigerated) and 40 °C (dry oven) for 48 h [31,32]. The samples were then visually inspected for phase separation, creaming, caking, and aggregation. Changes in the particle size (nm) and PDI were measured using a Zetasizer, and the transmittance (%) was measured using a UV–visible spectrophotometer (UV-1800, SHIMADZU, Kyoto, Japan).

### 2.11. In Vitro and Ex Vivo Diffusion Tests

The membrane used in the in vitro test was a 12,400 Da cellulose membrane for a Franz diffusion cell dialysis tube [18,29]. The membrane used in the ex vivo test was a bovine cornea [30]. Bovine eyes were collected immediately after slaughter from a local slaughterhouse and transported to the laboratory in PBS at 4 °C. Bovine corneas were carefully excised from the surrounding sclera, washed with 0.9% sodium chloride, and immediately inserted into Franz diffusion cells with the epithelial surface facing the donor and the endothelium facing the receptor [33]. Each membrane was positioned between the two compartments of the individual Franz cells (with spherical joints) so that the corneal epithelium faced the donor compartment containing either the test or control formulation. A drug dispersion was prepared by dispersing FLU in 0.5% carboxymethyl cellulose to a concentration of 242 µg/mL. A drug dispersion of 0.35 mL was used as the control group, whereas the experimental groups used FLU-SNE at a concentration of 242 µg/mL. The receptor compartment was filled with 15 mL of freshly prepared PBS (pH 7.4), and the system was maintained at 32 ± 0.2 °C with continuous stirring. The available diffusion area was 1.77 cm^2^. Samples were collected from the receptor compartment and replaced with an equal volume of fresh medium at specific time intervals. Samples from the 12,400 Da cellulose membrane were collected at 5-, 15-, 30-, 45-, 60-, 90-, and 120-min intervals, and samples from the bovine cornea were collected at 5-, 20-, 40-, 60-, 90-, 120-, 180-, 240-, 300-, and 360-min intervals [29]. The sample analysis was conducted using an HPLC system. The cumulative drug permeation (*Q_n_*) and steady-state flux (*J_ss_*) were calculated.

$$Q_{n}=\sum \left(C_{n}*V_{n}\right)$$

Cumulative drug permeation represents the cumulative amount of a drug transported over time. In the equation, *n* is the sampling time point, *C_n_* is the drug concentration at that time point, and *V_n_* is the volume of the solution (15.0 mL in this study) at the corresponding time point.

$$J_{ss}=\left(\frac{dQ}{dt}\right)/A$$

The steady-state flux was calculated using the rate of change in the cumulative drug transport per unit surface area. In the equation, *dQ*/*dt* represents the rate of change in the cumulative drug transport per unit of time. *A* is the effective area for drug permeation (1.77 cm^2^).

## 3. Results

### 3.1. Solubility of FLU

**Figure 1:**
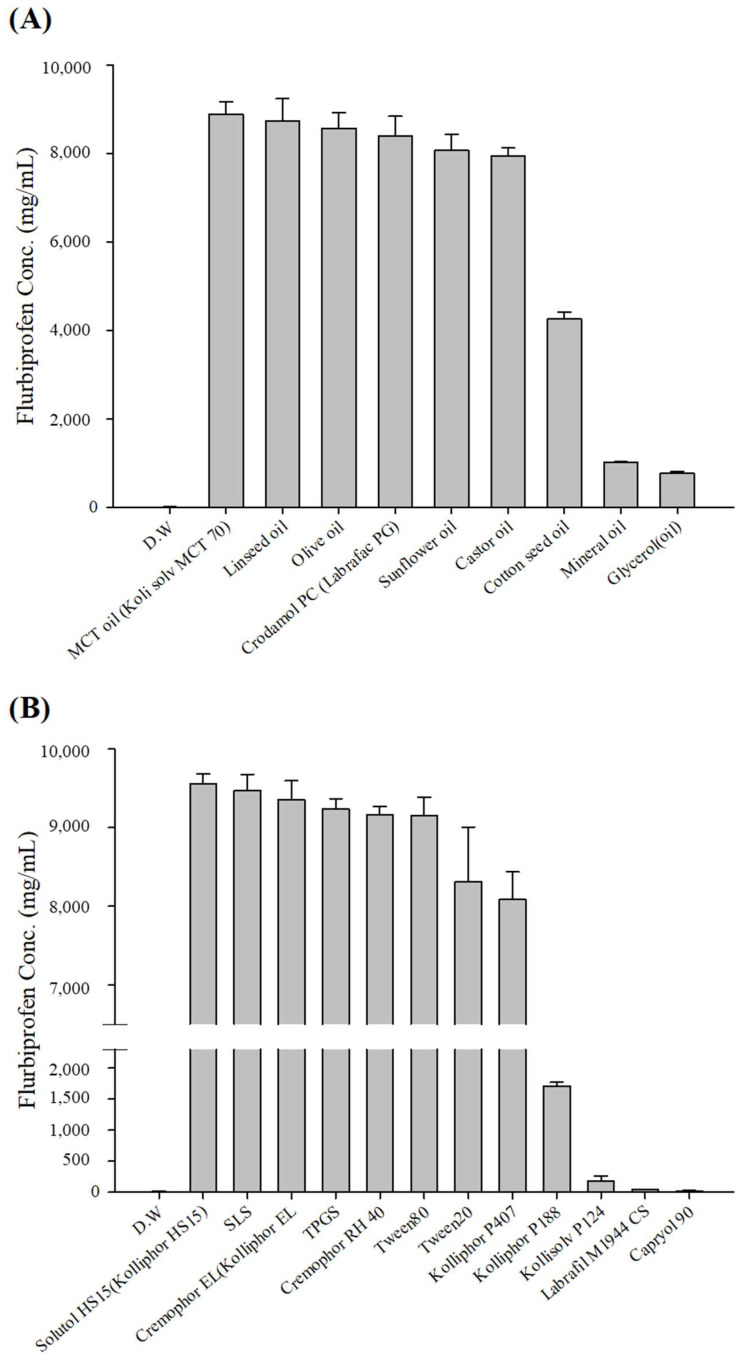
Solubility of FLU in various (**A**) oils and (**B**) 10% (*w*/*v*) surfactants.

The solubility of FLU was measured in an oil, a surfactant, and a co-surfactant. FLU showed the highest solubility in Kollisolv MCT 70 (8886.3 ± 281.1 μg/mL) (Figure 1). The stability of an emulsion can be increased using surfactants with high HLB values that have a strong affinity for hydrophilic parts and surfactants with low HLB values that have a strong affinity for hydrophobic parts [34]. In this study, we categorized the surfactants according to their HLB values. Solutol HS 15 (9558.7 ± 125.5 μg/mL), which has the highest solubility among surfactants with HLB values above 10, was used as the surfactant. Labrafil M1944CS (39.4 ± 9.6 μg/mL), which has good miscibility among surfactants with HLB values below 10, was used as the co-surfactant.

### 3.2. Pseudo-Ternary Phase Diagrams

**Figure 2:**
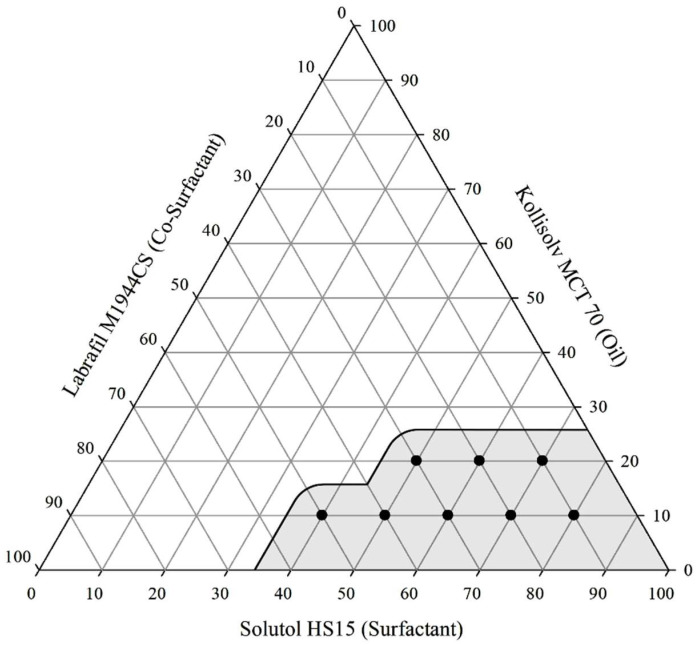
Pseudo-ternary diagram of Kollisolv MCT 70, Solutol HS 15, and Labrafil M1944CS (*w*/*w*/*w*).

Based on the solubility results in Section 3.1, the selected oil (Kollisolv MCT 70), surfactant (Solutol HS 15), and co-surfactant (Labrafil M1944CS) were used in a pseudo-ternary diagram to visually identify liquid SNEDDS formulations that could spontaneously form in water without phase separation or droplet aggregation. The shaded area in the diagram highlights the formulations with good self-nanoemulsification ability. Regions that displayed effective dispersion and emulsion formation were labeled as ‘good’ and colored gray, whereas regions that showed poor dispersion, agglomeration, or cream formation in oil were labeled as ‘bad’ and colored white (Figure 2) [35]. An analysis of the pseudo-ternary phase diagram showed that improved emulsions were generated when a higher ratio of Solutol HS 15 was used as a surfactant compared with Kollisolv MCT 70. This effect is mentioned in several studies. As the proportion of surfactant increases, the interface of the emulsion becomes more stabilized and condensed [36].

### 3.3. Optimization of the FLU-SNE Formulation Using a Box–Behnken Design (BBD)

FLU-SNE was optimized using Minitab 19^®^ and a BBD as the DoE (Design of Experiments). The 15 experimental designs included a center point and were used to understand the influence of the independent variables on dependent variables such as the particle size (Y_1_), PDI (Y_2_), zeta potential (Y_3_), and transmittance (Y_4_) [37]. A smaller particle size increases the interfacial surface area for drug absorption, and a lower PDI value indicates greater uniformity in the particle size distribution of the SNEDDS. The zeta potential represents the absence of globule aggregation in the continuous phase and serves as an indicator of non-coagulation efficiency, and higher transmittance indicates the formation of a transparent microemulsion. Therefore, the four factors are all critical characteristics of an SNEDDS and have a significant impact on ocular permeability [38,39]. Each response was selected to analyze the particle size (Y_1_), PDI (Y_2_), zeta potential (Y_3_), and transmittance (Y_4_), which are important factors in an SNEDDS. The models were selected based on significant *p*-values obtained from the ANOVA for each response variable [22,40]. The results obtained from all experiments are presented in Table 2.

#### 3.3.1. Effect of Variables on Particle Size (Y_1_)

The R^2^ value in the analysis of variance for particle size (Y_1_) was 96.75%, indicating a high explanatory power (Table 3). All linear model terms (X_1_, X_2_, X_3_) were significant as their *p*-values were below 0.05. In the quadratic model, X_1_^2^ and X_2_^2^ were also significant with *p*-values below 0.05, whereas X_3_^2^ was not significant with a *p*-value of 0.506. In the 2FI model, all interaction terms (X_1_X_2_, X_1_X_3_, X_2_X_3_) were significant as their *p*-values were below 0.05. The equation of the model in coded terms is presented as follows:

Y_1_ (particle size) = 36.66 + 8.38X_1_ − 21.07X_2_ + 16.00X_3_ + 6.60X_1_^2^ + 11.47X_2_^2^ − 0.96X_3_^2^ − 8.91X_1_X_2_ + 8.76X_1_X_3_ − 9.54X_2_X_3_

**Figure 3:**
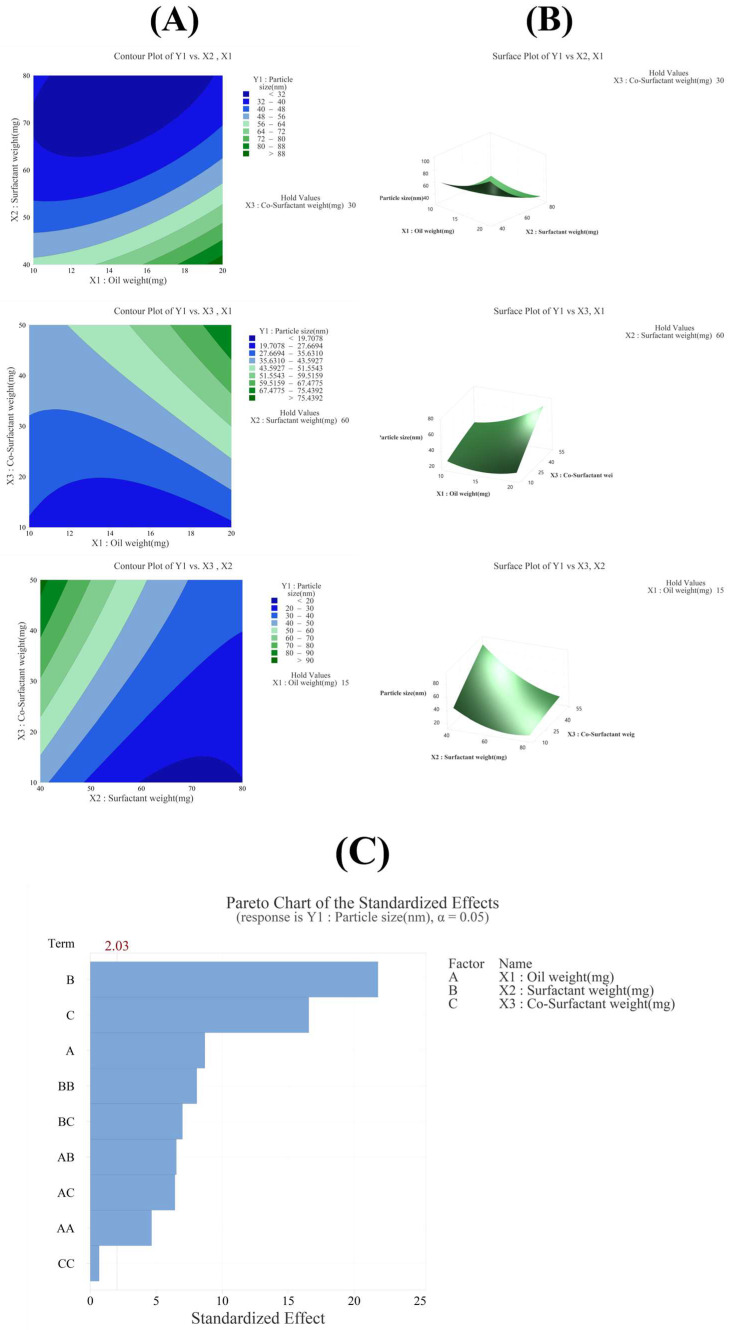
(**A**) Contour plot, (**B**) surface plot, and (**C**) Pareto chart for Y_1_ particle size.

The X_2_ factor had the greatest influence on particle size, as confirmed by the Pareto chart in Figure 3C, contour plot and surface plot in Figure 3B, and *p*-value in Table 3. In addition, the Pareto chart in Figure 3C indicated that factors X_2_, X_3_, and X_1_ influenced particle size, in that order, demonstrating that these three factors contributed significantly to determining the particle size of the SNEDDS [22,23,35,40,41].

#### 3.3.2. Effect of Variables on PDI (Y_2_)

The R^2^ value in the analysis of variance for the PDI (Y_2_) was 91.78%, indicating a high explanatory power. In the linear model, X_1_ was not significant as its *p*-value was 0.450, whereas X_2_ and X_3_ were significant with *p*-values below 0.05. In the quadratic model, X_1_^2^ was significant with a *p*-value below 0.05, but X_2_^2^ and X_3_^2^ were not significant, with *p*-values of 0.182 and 0.634, respectively. In the 2FI model, X_1_X_2_ and X_2_X_3_ were not significant, with *p*-values of 0.230 and 0.104, respectively, whereas X_1_X_3_ was significant with a *p*-value below 0.05. The equation of the model in coded terms is presented as follows:

Y_2_ (PDI) = 0.12 + 0.00X_1_ − 0.05X_2_ + 0.04X_3_ + 0.03X_1_^2^ + 0.01X_2_^2^ − 0.00X_3_^2^ + 0.01X_1_X_2_ + 0.03X_1_X_3_ − 0.01X_2_X_3_

**Figure 4:**
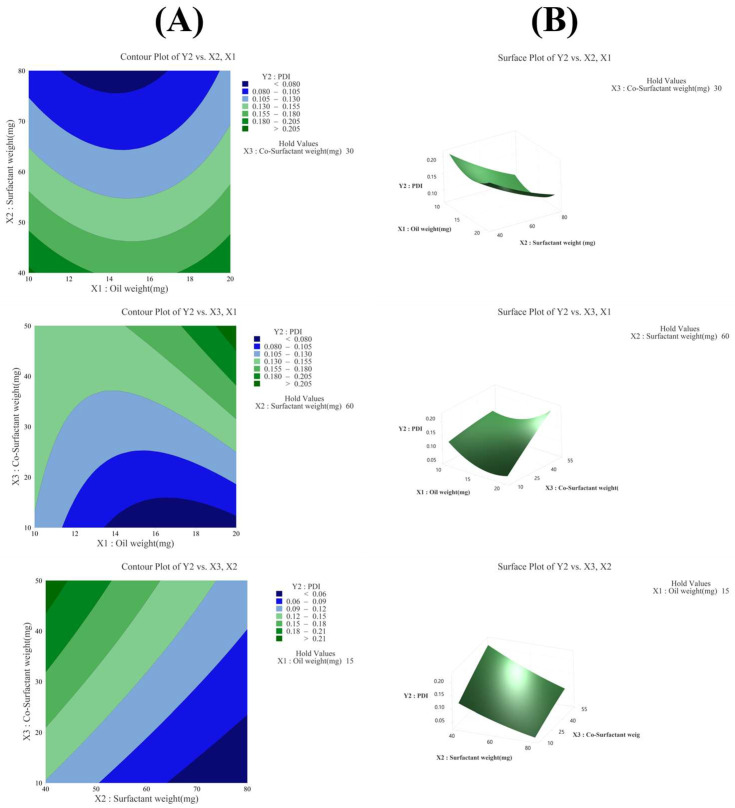

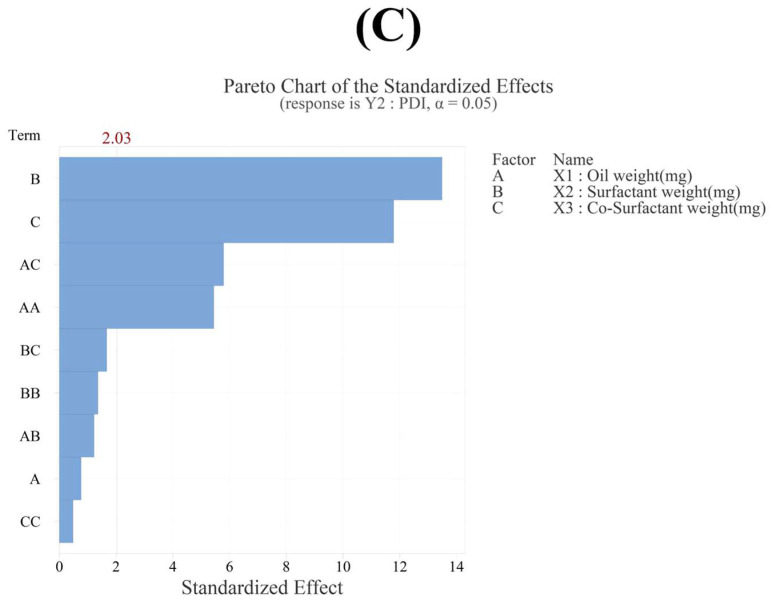
(**A**) Contour plot, (**B**) surface plot, and (**C**) Pareto chart for Y_2_ PDI.

The X_2_ factor had the greatest influence on the PDI, as confirmed by the Pareto chart in Figure 4C, contour plot and surface plot in Figure 4B, and *p*-value in Table 4. As shown in the Pareto chart in Figure 4C, it was statistically confirmed that only the factors X_2_, X_3_, X_1_X_3_, and X_1_^2^ significantly affected the PDI.

#### 3.3.3. Effect of Variables on Zeta Potential (Y_3_)

The selected X factors did not have a significant effect on the zeta potential (Y_3_) values, resulting in a low R^2^ value of 35.53% in the analysis of variance. Therefore, the zeta potential (Y_3_) was excluded when optimization tools were used to select the optimized formulation.

#### 3.3.4. Effect of Variables on Transmittance (Y_4_)

The R^2^ value in the analysis of variance for transmittance (Y_4_) was 95.02%, indicating a high explanatory power (Table 5). All linear model terms (X_1_, X_2_, X_3_) were significant as their *p*-values were below 0.05. In the quadratic model, X_1_^2^ and X_2_^2^ were significant with *p*-values below 0.05, whereas X_3_^2^ was not significant with a *p*-value of 0.687. In the 2FI model, X_1_X_2_ was not significant with a *p*-value of 0.348, whereas X_2_X_3_ and X_1_X_3_ were significant with *p*-values below 0.05. The equation of the model in coded terms is presented as follows:

Y_4_ (transmittance) = 61.20 − 6.02X_1_ + 16.88X_2_ − 11.02X_3_ − 4.81X_1_^2^ − 5.85X_2_^2^ − 0.64X_3_^2^ − 1.43X_1_X_2_ − 16.81X_1_X_3_ + 17.26X_2_X_3_

**Figure 5:**
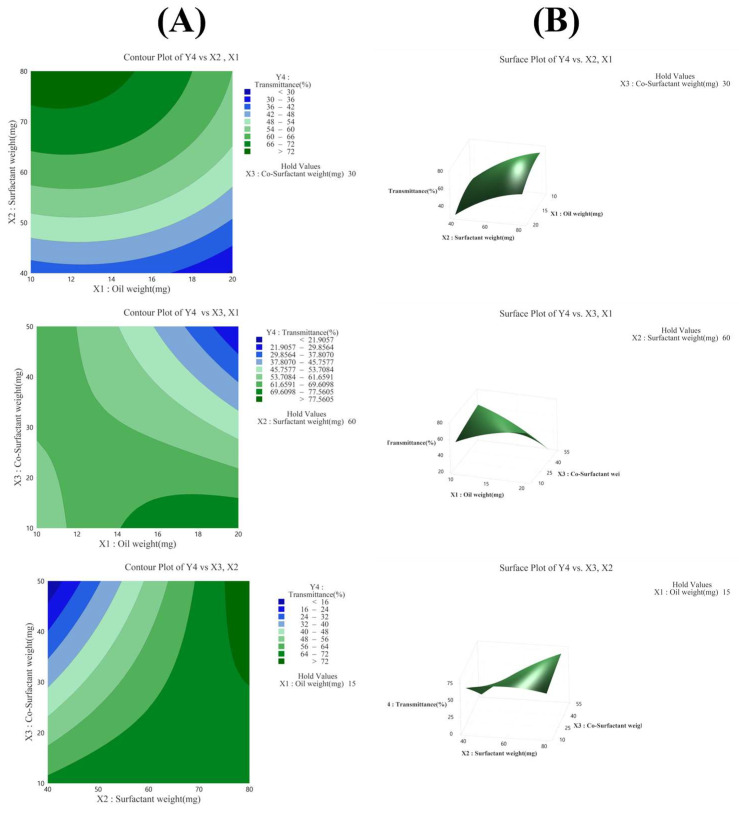

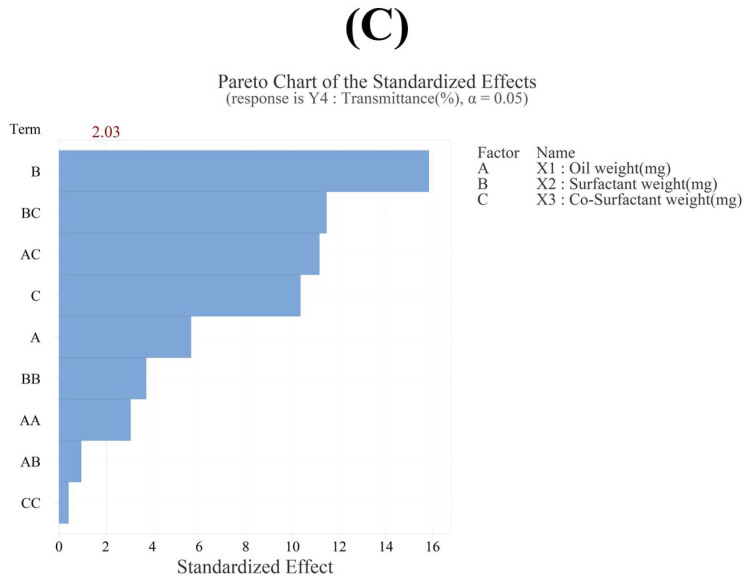
(**A**) Contour plot, (**B**) surface plot, and (**C**) Pareto chart for Y_4_ transmittance.

The X_2_ factor had the greatest influence on transmittance, as confirmed by the Pareto chart in Figure 5C, contour plot and surface plot in Figure 5B, and *p*-value in Table 5. In addition, the Pareto chart in Figure 5C indicated that factors X_2_, X_2_X_3_, X_1_X_3_, and X_3_ influenced the particle size, in that order, demonstrating that these four factors contributed significantly to determining the particle size of the SNEDDS [22,23,35,40,41].

### 3.4. Characterization of Optimized FLU-SNE

**Figure 6:**
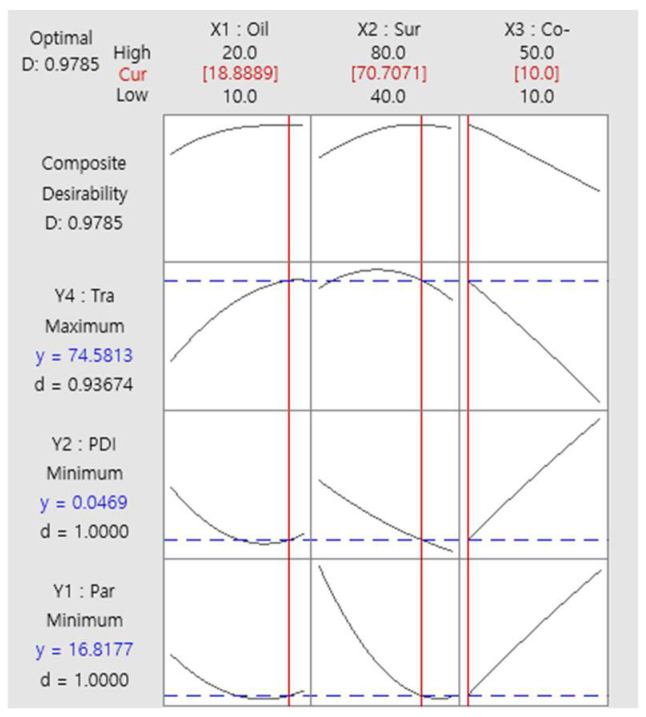
Optimized process variables (desirability function analysis).

**Figure 7:**
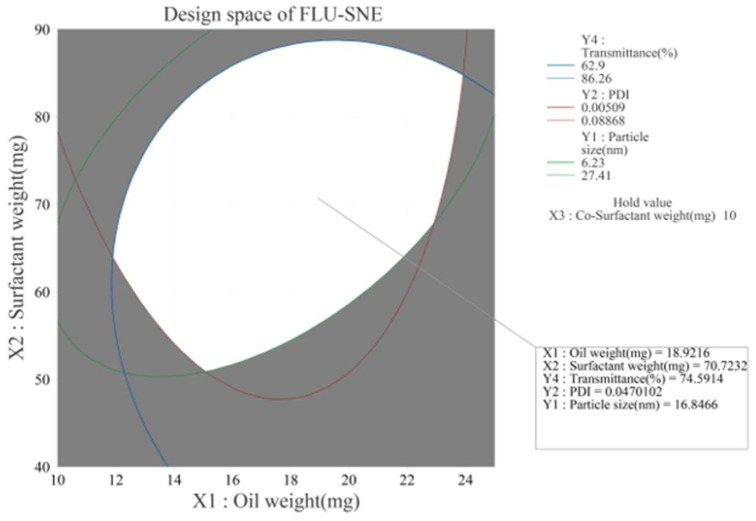
Overlaid contour plots depicting the design space.

In multiple-response optimization, the desirability function approach is commonly used. To optimize multiple responses simultaneously, it is desirable to consider conflicting criteria and incorporate the decision maker’s preferences. Generally, the overall desirability D-value (D) is considered higher when it is closer to 1 [42,43]. A response optimization tool was used to optimize the factors analyzed in the BBD. The optimization process aimed to identify the highest transmittance, lowest PDI, and smallest particle size [37]. Optimization was conducted using an optimization tool, and the optimization results were as follows: X_1_: oil weight 18.9 mg, X_2_: surfactant weight 70.7 mg, and X_3_: co-surfactant weight 10.0 mg (Table 6; Figure 6). In the BBD study of formulation optimization, the derived response surface model elucidated the interplay among three critical formulation parameters. These were the oil weight (X_1_), surfactant weight (X_2_), and co-surfactant weight (X_3_) (Figure 6). The D-value of composite preference was 0.9785, close to 1, which represented the optimal combination of factors within the studied parameter space (Figure 6) [43,44]. The design space is a multidimensional region that includes all independent and dependent variables [26,27]. The design space plot represents the effects of X_1_ (oil) and X_2_ (surfactant) on the responses of Y_1_ (particle size), Y_2_ (PDI), and Y_3_ (transmittance) when factor X_3_ (co-surfactant) was fixed at 10 mg (Figure 7) [29]. The design space was represented by a white area on the plot, which signified the range of variable levels that conformed to the optimal product characteristics (Figure 7).

### 3.5. Evaluation of Optimized FLU-SNE

#### 3.5.1. Evaluation of Reproducibility of Optimized FLU-SNE

**Figure 8:**
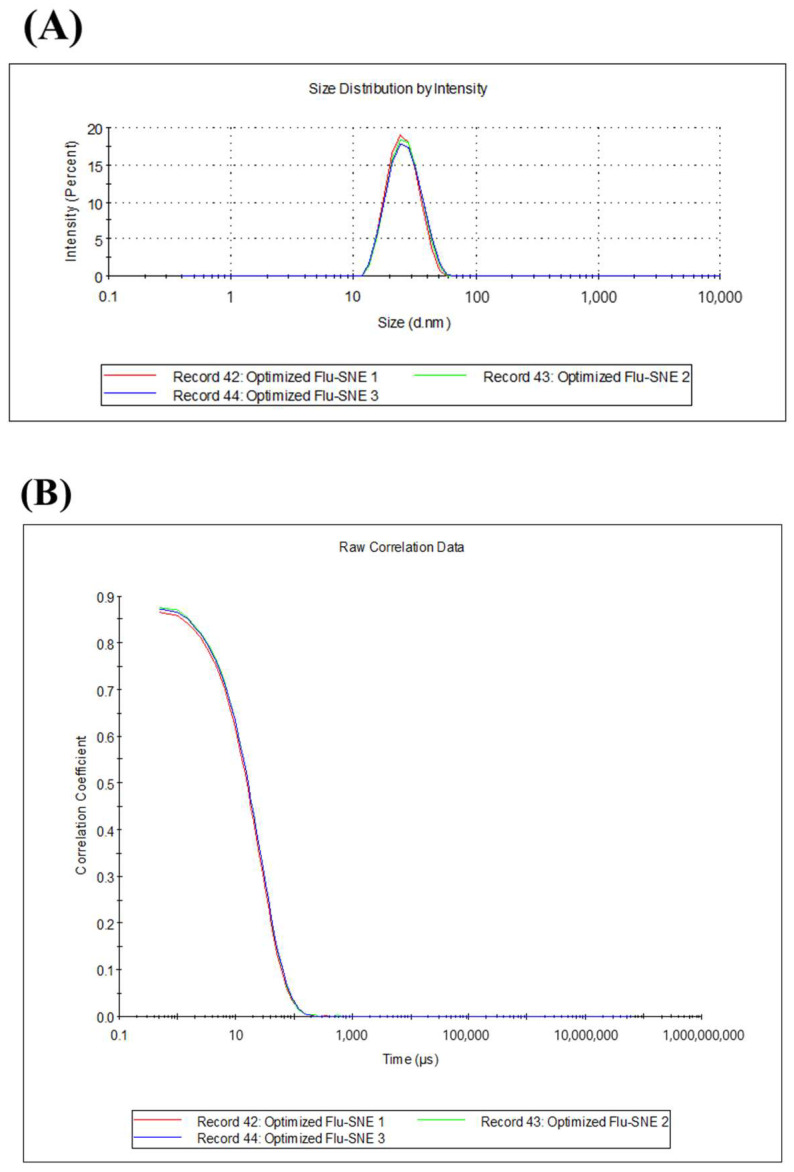
Optimized FLU-SNE reproducibility (particle size): (**A**) monovariance graph and (**B**) correlation graph.

The reproducibility results for the optimized FLU-SNE were 24.89 nm for Y_1_ (particle size), 0.068 for Y_2_ (PDI), and 74.85% for Y_4_ (transmittance) (Table 7). The optimized FLU-SNE was used after 10-fold dilution with PBS. The results indicated that Y_1_ was 24.89 nm, which was within the 95% prediction interval (PI) of the fitted value shown in the response optimizer results. Y_2_ was 0.068, which was also within the 95% PI of the fitted value. Y_3_ was 74.85%, which was within the 95% PI of the fitted value [45]. FLU-SNE met the manufacturing target quality because all measurements were within the 95% prediction intervals. Figure 8 depicts the dynamic light scattering measurement results for the optimized formulation, where (A) illustrates the measurement results in the form of monodispersity, and (B) depicts the SNEDDS sample as a stable nanoemulsion with a uniformly distributed particle size and consistent behavior across multiple measurements [46].

#### 3.5.2. Thermodynamic Stability of FLU-SNE

**Figure 9:**
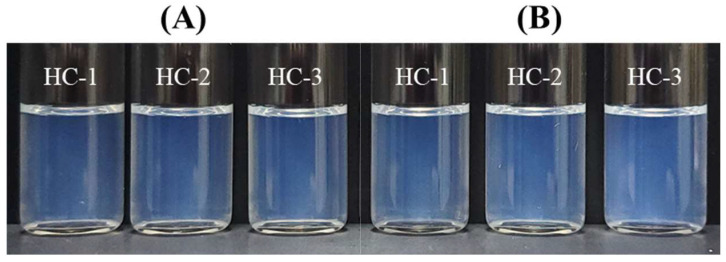
Observation of creaming and caking phase separations for FLU-SNE: (**A**) initial results and (**B**) after 48 h.

An SNEDDS must be stable under a wide range of temperature conditions and must not cause system collapse upon dilution. We performed thermodynamic stability studies using the method identified in Section 2.9. No creaming, caking, or phase separation was observed between pre- and post-conditions before and after six cycles of 40 °C to 4 °C (Figure 9). The particle size was 26.63 nm, the PDI was 0.088, and the transmittance was 72.90%, with no difference between pre- and post-conditions. Therefore, we ascertained that FLU-SNE was thermodynamically stable [31,32].

#### 3.5.3. pH of FLU-SNE

pH is an important factor in ophthalmic formulations. The FLU-SNE pH test result was 7.16 ± 0.01, a pH that does not cause local eye pain [47].

#### 3.5.4. Morphological Evaluation of FLU-SNE

**Figure 10:**
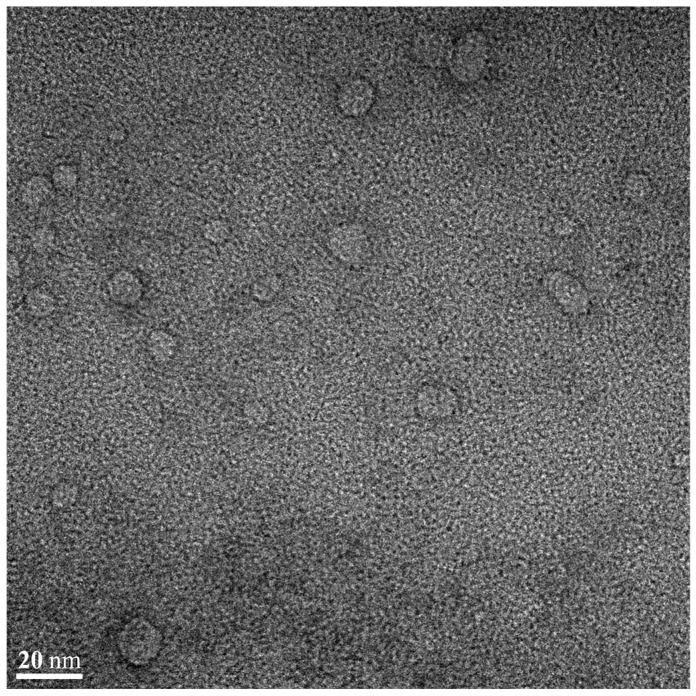
Transmission electron microscopy image of optimized FLU-SNE (20 nm).

Figure 10 shows a representative TEM image of FLU-SNE dispersed in a buffer with a pH of 6.8. FLU-SNE was observed to contain single spherical and translucent nanoemulsion droplets with a mean diameter of <25 nm. This value was comparable with that obtained when the particle size was analyzed using dynamic light scattering.

### 3.6. In Vitro Diffusion Test

**Figure 11:**
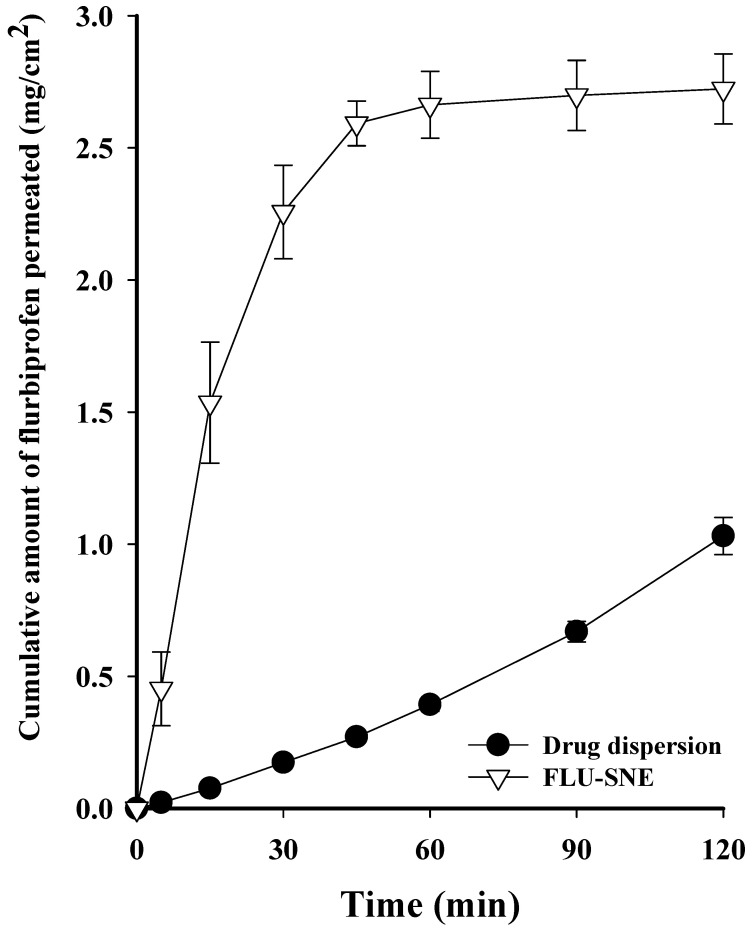
In vitro release profile of FLU-SNE and drug dispersion.

The flux of DQS in the 12,400 Da cellulose membrane was observed to be 2.723 ± 0.133 (mg/cm^2^) for FLU-SNE and 1.031 ± 0.070 (mg/cm^2^) for the drug dispersion, indicating that FLU-SNE exhibited approximately 2.5 times higher flux than the drug dispersion. FLU-SNE has a small particle size, which increases the interfacial surface area for drug absorption, and has a low PDI value, which provides good uniformity and higher permeability compared to drug dispersions [11,12,16]. Eye drops are rapidly eliminated from the eye, and the higher the initial penetration of a drug, the better its absorption. [16,48,49]. FLU-SNE has higher initial penetration and maximum flux than a drug dispersion, which is advantageous for drug absorption in ophthalmic formulations (Figure 11) [13,49].

### 3.7. Ex Vivo Diffusion Test

**Figure 12:**
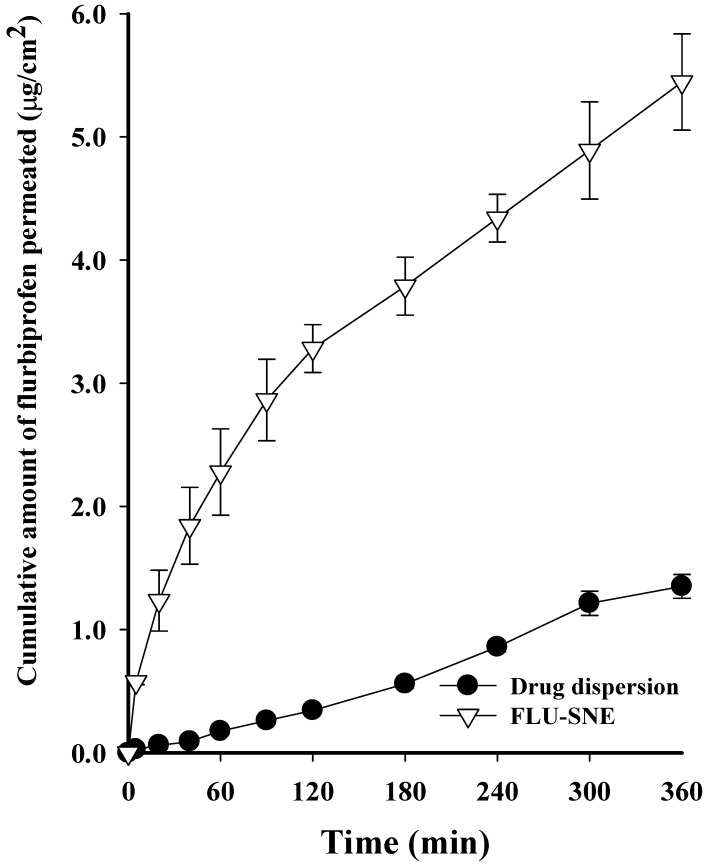
Ex vivo release profile of FLU-SNE and drug dispersion.

The flux of DQS in the bovine cornea was observed to be 5.446 ± 0.390 (μg/cm^2^) for FLU-SNE and 1.350 ± 0.097 (μg/cm^2^) for the drug dispersion, indicating that FLU-SNE exhibited approximately 4 times higher flux than the drug dispersion. FLU-SNE has a small particle size, which increases the interfacial surface area for drug absorption, and has a low PDI value, which provides good uniformity and higher permeability compared to drug dispersions [11,12,16]. The drug diffusion pattern was higher in the early stage; this is similar to the in vitro drug diffusion results, which were initially higher than those for the drug dispersion [16,48,49]. FLU-SNE had higher initial penetration and maximum flux than the drug dispersion, which is advantageous for drug absorption in ophthalmic formulations (Figure 12) [16,49]. The in vitro and ex vivo diffusion test results commonly showed faster and higher penetration for FLU-SNE than for the drug dispersion. This suggests that FLU-SNE increases the initial penetration and maximum penetration of drugs in ophthalmic formulations, which is expected to enhance drug bioavailability [13,49].

## 4. Conclusions

In this study, we developed and optimized an SNEDDS for the topical ocular administration of FLU using a QbD approach. Using a BBD, we optimized a formulation with a small particle size, low PDI, and high transmittance by considering the oil weight, surfactant weight, and co-surfactant weight, which are factors of SNEDDSs, as variables. The morphological test results supported the DLS results, and FLU-SNE exhibited good thermodynamic stability without phase separation or creaming, even under stress conditions such as heating–cooling cycles. The permeability test on FLU-SNE using a Franz diffusion cell confirmed that the drug flux was approximately 2.5 times higher in in vitro experiments using a cellulose membrane, and about 4 times higher in ex vivo experiments using excised bovine corneal tissue, compared to that of a drug dispersion. This suggests that the FLU-SNE developed in this study can significantly improve drug delivery efficiency as a topical ocular administration formulation. Although there have been many studies on SNEDDS formulations using existing FLU, most of them focused on oral administration systems or enhancing systemic absorption. Therefore, in this study, FLU with low solubility in water was optimized as FLU-SNE suitable for topical ocular administration using a QbD approach. The results regarding the reproducibility, stability, and permeability of FLU-SNE suggest the potential for more effective topical ophthalmic FLU-SNEDDSs.

## Figures and Tables

**Table 1:** Factor levels of the Box–Behnken design.

	Level	
Factor	−1 (Low)	+1 (High)
X_1_: Oil weight (mg)	10	20
X_2_: Surfactant weight (mg)	40	80
X_3_: Co-surfactant weight (mg)	10	50

**Table 2:** Factor levels and responses of the Box–Behnken design.

X_1_	X_2_	X_3_	Y_1_			Y_2_		
Oil (mg)	Surfactant (mg)	Co-Surfactant (mg)	Particle Size (nm)			PDI		
			1	2	3	1	2	3
20	80	30	34.38	32.98	32.33	0.100	0.089	0.091
20	60	50	72.67	70.12	69.98	0.237	0.231	0.239
20	60	10	23.16	22.58	22.56	0.071	0.047	0.047
20	40	30	105.40	99.55	97.09	0.214	0.213	0.226
15	80	50	37.93	36.62	36.41	0.123	0.110	0.101
15	80	10	23.71	22.34	22.14	0.061	0.072	0.053
15	60	30	38.08	36.55	36.30	0.111	0.114	0.111
15	60	30	35.01	35.31	35.25	0.114	0.117	0.120
15	60	30	38.80	37.49	37.14	0.135	0.110	0.111
15	40	50	93.57	90.06	88.48	0.204	0.200	0.189
15	40	10	39.20	38.01	37.63	0.112	0.117	0.108
10	80	30	27.61	26.24	25.96	0.067	0.082	0.083
10	60	50	45.34	44.01	43.65	0.159	0.16	0.174
10	60	10	32.30	30.82	30.49	0.111	0.116	0.116
10	40	30	59.42	58.08	57.76	0.229	0.230	0.225
**X_1_**	**X_2_**	**X_3_**	**Y_3_**			**Y_4_**		
**Oil** **(mg)**	**Surfactant** **(mg)**	**Co-Surfactant** **(mg)**	**Zeta Potential (mV)** **(Absolute Value)**			**Transmittance** **(%)**		
			**1**	**2**	**1**	**2**	**1**	**2**
20	80	30	1.310	0.444	1.310	0.444	1.310	0.444
20	60	50	4.220	6.270	4.220	6.270	4.220	6.270
20	60	10	3.270	1.250	3.270	1.250	3.270	1.250
20	40	30	0.483	0.113	0.483	0.113	0.483	0.113
15	80	50	2.580	0.286	2.580	0.286	2.580	0.286
15	80	10	0.178	0.951	0.178	0.951	0.178	0.951
15	60	30	1.880	2.390	1.880	2.390	1.880	2.390
15	60	30	2.150	3.050	2.150	3.050	2.150	3.050
15	60	30	0.373	0.258	0.373	0.258	0.373	0.258
15	40	50	0.275	0.215	0.275	0.215	0.275	0.215
15	40	10	0.766	3.840	0.766	3.840	0.766	3.840
10	80	30	0.893	0.600	0.893	0.600	0.893	0.600
10	60	50	1.270	1.800	1.270	1.800	1.270	1.800
10	60	10	0.724	1.410	0.724	1.410	0.724	1.410
10	40	30	5.820	2.900	5.820	2.900	5.820	2.900

**Table 3:** Y_1_ particle size ANOVA results and model summary.

	DF *	Adj SS *	Adj MS *	F-Value	*p*-Value
Model	9	23,321.3	2591.3	115.60	0.000
Linear Model	3	18,477.5	6159.2	274.77	0.000
X_1_: Oil (mg)	1	1685.4	1685.4	75.19	0.000
X_2_: Surfactant (mg)	1	10,651.3	10,651.3	475.17	0.000
X_3_: Co-Surfactant (mg)	1	6140.8	6140.8	273.95	0.000
Quadratic Model	3	1878.8	626.3	27.94	0.000
X_1_: Oil^2^	1	483	483.0	21.55	0.000
X_2_: Surfactant^2^	1	1457.6	1457.6	65.03	0.000
X_3:_ Co-Surfactant^2^	1	10.1	10.1	0.45	0.506
Two-Factor Interaction	3	2965	988.3	44.09	0.000
X_1_: Oil · X_2_: Surfactant	1	952.3	952.3	42.48	0.000
X_1_: Oil · X_3_: Co-Surfactant	1	920.2	920.2	41.05	0.000
X_2_: Surfactant · X_3_: Co-Surfactant	1	1092.5	1092.5	48.74	0.000
S	R^2^	R^2^ (Retouch)		R^2^ (Prediction)	
4.73451	96.75%	95.91%		94.24%	

* DF: Degrees of Freedom, Adj SS: Adjusted Sum of Squares, Adj MS: Adjusted Mean Squares.

**Table 4:** Y_2_ PDI ANOVA results and model summary.

	DF	Adj SS	Adj MS	F-Value	*p*-Value
Model	9	0.136459	0.015162	43.43	0.000
Linear Model	3	0.112265	0.037422	107.18	0.000
X_1_: Oil (mg)	1	0.000204	0.000204	0.58	0.450
X_2_: Surfactant (mg)	1	0.063551	0.063551	182.03	0.000
X_3_: Co-Surfactant (mg)	1	0.048510	0.048510	138.94	0.000
Quadratic Model	3	0.010983	0.003661	10.49	0.000
X_1_: Oil^2^	1	0.010342	0.010342	29.62	0.000
X_2_: Surfactant^2^	1	0.000646	0.000646	1.85	0.182
X_3:_ Co-Surfactant^2^	1	0.000080	0.000080	0.23	0.634
Two-Factor Interaction	3	0.013211	0.004404	12.61	0.000
X_1_: Oil · X_2_: Surfactant	1	0.000520	0.000520	1.49	0.230
X_1_: Oil · X_3_: Co-Surfactant	1	0.011719	0.011719	33.57	0.000
X_2_: Surfactant · X_3_: Co-Surfactant	1	0.000972	0.000972	2.78	0.104
S	R^2^	R^2^ (Retouch)		R^2^ (Prediction)	
0.0186851	91.78%	89.67%		85.56%	

**Table 5:** Y_4_ transmittance ANOVA results and model summary.

	DF	Adj SS	Adj MS	F-Value	*p*-Value
Model	9	18,204.7	2022.75	74.21	0.000
Linear Model	3	10,620.8	3540.28	129.88	0.000
X_1_: Oil (mg)	1	870.1	870.11	31.92	0.000
X_2_: Surfactant (mg)	1	6838.0	6838.02	250.86	0.000
X_3_: Co-Surfactant (mg)	1	2912.7	2912.72	106.86	0.000
Quadratic Model	3	591.2	197.08	7.23	0.001
X_1_: Oil^2^	1	256.1	256.06	9.39	0.004
X_2_: Surfactant^2^	1	379.5	379.46	13.92	0.001
X_3:_ Co-Surfactant^2^	1	4.5	4.49	0.16	0.687
Two-Factor Interaction	3	6992.6	2330.88	85.51	0.000
X_1_: Oil · X_2_: Surfactant	1	24.7	24.71	0.91	0.348
X_1_: Oil · X_3_: Co-Surfactant	1	3391.3	3391.28	124.41	0.000
X_2_: Surfactant · X_3_: Co-Surfactant	1	3576.7	3576.65	131.21	0.000
S	R^2^	R^2^ (Retouch)		R^2^ (Prediction)	
5.22092	95.02%	93.74%		91.15%	

**Table 6:** Multiple-response predictions.

Factor			Setting	
X_1_: Oil Weight (mg)			18.9	
X_2_: Surfactant Weight (mg)			70.7	
X_3_: Co-Surfactant Weight (mg)			10.0	
**Response**	**Suitable Value**	**SE Suitable Value**	**95% CI**	**95% PI**
Y_1_: Particle Size (nm)	16.82	2.19	(12.37, 21.26)	(6.23, 27.41)
Y_2_: PDI	0.047	0.009	(0.029, 0.064)	(0.005, 0.089)
Y_4_: Transmittance (%)	74.58	2.42	(69.68, 79.48)	(62.90, 86.26)

**Table 7:** Evaluation of optimized FLU-SNE.

Particle Size (nm)	PDI	Transmittance (%)
24.89 ± 0.28	0.068 ± 0.008	74.85 ± 5.69

## Data Availability

Data available on request due to restrictions, e.g., privacy or ethical restrictions.
